# The role of prickle proteins in vertebrate development and pathology

**DOI:** 10.1007/s11010-023-04787-z

**Published:** 2023-06-26

**Authors:** K. A. Radaszkiewicz, M. Sulcova, E. Kohoutkova, J. Harnos

**Affiliations:** Department of Experimental Biology, Faculty of Science, Masaryk University, Brno, 62500 Czechia

**Keywords:** Prickle, Planar cell polarity (PCP), Vertebrates, Embryonic development, Pathology

## Abstract

Prickle is an evolutionarily conserved family of proteins exclusively associated with planar cell polarity (PCP) signalling. This signalling pathway provides directional and positional cues to eukaryotic cells along the plane of an epithelial sheet, orthogonal to both apicobasal and left–right axes. Through studies in the fruit fly *Drosophila*, we have learned that PCP signalling is manifested by the spatial segregation of two protein complexes, namely Prickle/Vangl and Frizzled/Dishevelled. While Vangl, Frizzled, and Dishevelled proteins have been extensively studied, Prickle has been largely neglected. This is likely because its role in vertebrate development and pathologies is still being explored and is not yet fully understood. The current review aims to address this gap by summarizing our current knowledge on vertebrate Prickle proteins and to cover their broad versatility. Accumulating evidence suggests that Prickle is involved in many developmental events, contributes to homeostasis, and can cause diseases when its expression and signalling properties are deregulated. This review highlights the importance of Prickle in vertebrate development, discusses the implications of Prickle-dependent signalling in pathology, and points out the blind spots or potential links regarding Prickle, which could be studied further.

**Supplementary Information:**

The online version contains supplementary material available at 10.1007/s11010-023-04787-z.

## Introduction

Prickle, originally discovered in *Drosophila* in the 1940s, gets its name from a gene mutant phenotype with disoriented thoracic bristles, described as “irregularly erected and whorled, giving a *prickle* effect “ [1]. This phenotype has been linked to disrupted WNT/planar cell polarity (PCP) signalling responsible for forming and orienting body surface structures [2]. As this mutant fly was not lethal, Prickle might have been assumed to be of lesser importance for invertebrate signalling. PCP signalling complexity in vertebrates, however, extended to dynamic and more sophisticated events such as neural tube formation, organogenesis, and cell migration [3]. This is evidenced by the fact that the single invertebrate Prickle protein is duplicated into four paralogs in vertebrates, Prickle1-4 (PRICKLE1-4 in humans), identified in the late 1990s and early 2000s [4–8].

**Fig. 1:**
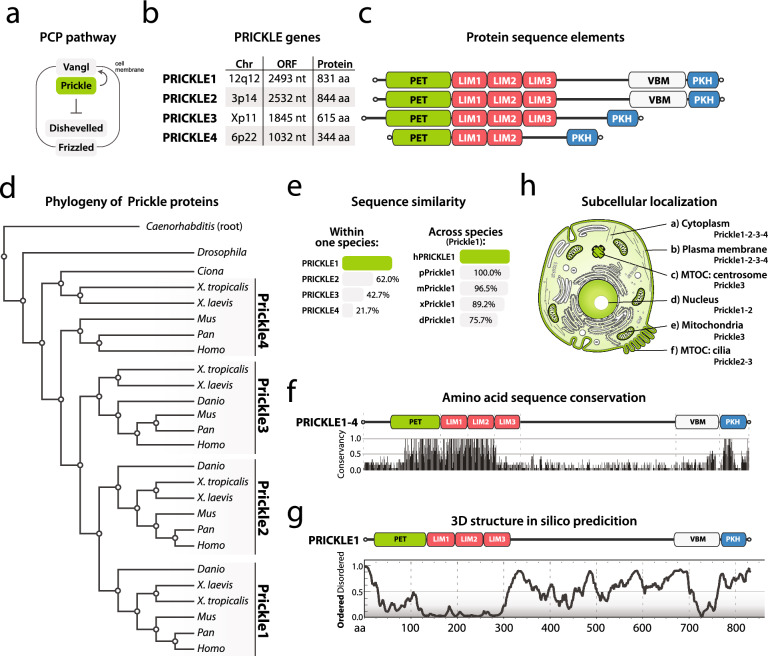
**a** Basic PCP pathway components and their subcellular distribution. **b** The human *PRICKLE1-4* gene locations and basic info. **c** Sequence elements of human PRICKLE1-4. For single sequence element description and abbreviations, see the accompanying text. **d** Phylogenetic analysis the of vertebrate Prickle family. Uniprot protein database was used to search for Prickle family members from main vertebrate species, which often serve as model organisms like *Homo Sapiens*, *Pan Troglodytes*, *Mus musculus*, *Xenopus laevis*, *Xenopus tropicalis*, and *Danio rerio*. We also added invertebrate species such as *Ciona intestinalis*, *Drosophila melanogaster*, and *Caenorhabditis elegans* as out-grouping to construct a phylogenetic tree. After collecting the relevant protein sequences, we used the MUSCLE algorithm to align the amino acid sequences and then used the Maximum Likelihood method to construct a phylogenetic tree. The tree shows the relationship of vertebrate Prickle1-4. A detailed description can be found in Data availability. **e** Amino acid sequence conservation in human PRICKLE1-4, compared to PRICKLE1; and individual combinations with Prickle1 isoforms across vertebrates, compared to human PRICKLE1. **f** Single amino acid sequence conservation in human PRICKLE1-4 showing the most conserved amino acids. **g** 3D structure in silico prediction for human PRICKLE1 using PONDR-Fit. Score 0.0–0.5 (the bottom part) means that the region forms the secondary structure, score 0.5–1.0 (the upper part) identifies a disordered region. **h** Prickle protein schematized depiction of eukaryotic cells showing six described subcellular locations of s

Functionally, Prickle is a cytoplasmic protein with no known enzymatic activity and plays an essential part in the PCP mechanism [2, 3, 9]. To fulfil its function, Prickle binds to the four-span transmembrane protein Vangl [10], resulting in the accumulation of Vangl-Prickle complexes at the plasma membrane, where it primarily regulates the actin cytoskeleton (Fig. 1a) [11, 12]. In addition, Prickle proteins inhibit other PCP proteins such as cytoplasmic Dishevelled [10] and its transmembrane binding partner Frizzled that form the opposite PCP complex (Fig. 1a) [2, 3, 9]. Both of these Prickle activities are necessary for PCP signalling, and this phenomenon is reviewed elsewhere [2, 3, 9].

Our review is centred on three vertebrate Prickle protein aspects. First, we discuss Prickle protein sequence elements, similarity, and subcellular localization. Second, we will explore the crucial role of Prickle proteins in the development of vertebrates. In the early vertebrate embryo, Prickle proteins are known to have roles in gastrulation and body axis formation. During neurulation and later development, Prickle is involved in, but not limited to, cell polarization in various tissues and organogenesis. Third, this review seeks to uncover the impact of PRICKLE proteins on maintaining tissue homeostasis and the progression of diseases in humans. As PRICKLE protein levels and function can be altered in tumours, special emphasis will be placed on their role in cancer.

Overall, Prickle proteins are implicated in a wide range of physiological processes, as well as pathological processes in vertebrates. Hence, appreciating the multifaceted roles of Prickle is of great importance to our understanding of the physiological development and progression of pathologies.

## Prickle protein sequence elements, similarity, and subcellular localization

### Sequence elements

The human *PRICKLE* gene family consists of four members, each encoding a protein of distinct length located on different chromosomes (Fig. 1b). All PRICKLE protein isoforms are modular and contain three sequence elements, the N-terminal PET and LIM domains, and the C-terminal PKH domain (Fig. 1c; **Suppl. Figure 1**).

**PET domain**. The PET (Prickle, Espinas, Testin) domain is found in Prickle, LIM-9, and Testin proteins (Espinas is an alternate name for Prickle2) and comprises ~ 110 amino acids, forming several α-helices [13, 14]. It is involved in both signal transduction and protein–protein interactions implicated in a variety of cellular processes such as cell adhesion, migration, and differentiation [13]. These cellular processes are typical for vertebrate Prickle paralogs, as described further. If the PET domain is accompanied by LIM domains like in Prickle proteins, its membrane binding potential is increased [14], which is an important prerequisite for PCP signalling.

**LIM domain**. The LIM (Linl-1, Isl-1, Mec-3) domain is an evolutionarily conserved cysteine-rich protein module composed of ~ 60 amino acids and is found in a wide variety of proteins collectively known as LIM proteins [15]. A single LIM domain consists of two zinc fingers, which are two antiparallel β-hairpin structures, separated by a two-amino acid hydrophobic linker residue. The LIM domain is involved in many cellular processes, from gene transcription to cytoskeleton organization. Moreover, this domain acts as an adaptor, mediating protein–protein interactions [16]. LIM domain-containing proteins often shuttle between the nucleus, where they regulate gene expression, and the cytosol, where they interact with the actin cytoskeleton, namely structures such as focal adhesions and adherens junctions [16]. All these features are well reflected in Prickle proteins and their function, as described further.

The vertebrate Prickle1-3 paralogs contain three LIM domains (Fig. 1c; **Suppl. Figure 1**), and this applies also to invertebrates’ homologs (**Suppl. Figure 1**). However, vertebrate Prickle4 contains only two LIM domains (Fig. 1c; **Suppl. Figure 1**), and this might be the reason Prickle4 isoforms are not considered a *bona fide* Prickle family member by some researchers [17]. As the Prickle protein has several LIM domains, they allow for multiple binding sites, enabling the protein to interact with other molecules simultaneously. Then, each LIM domain is composed of different amino acid sequences, enabling the protein to interact with different types of molecules. Finally, three LIM domains provide flexibility in the protein's structure, allowing it to bind to multiple molecules in various orientations. To sum up, the presence of several LIM domains gives proteins the ability to better interact with their cellular environment [18].

**PKH domain**. The PKH (Prickle homology) domain is found only in the vertebrate Prickle protein family, based on our sequence-based database search. Neither is its function nor secondary structure well known [7], but this domain is assumed to be involved in Prickle membrane localization due to its terminal CAAX sequence. CAAX, a common protein-targeting motif found in many eukaryotic proteins, consists of a C-terminal tetrapeptide sequence generally described as having an invariant cysteine (C), two aliphatic amino acids (A_1_ and A_2_), and one of several amino acids in the terminal position (X). The CAAX motif is important for proteins’ post-translational modification (PTM), as it is recognized by a family of prenyltransferases that can add a farnesyl or geranylgeranyl group to the cysteine amino acid [19]. This modification allows the proteins to be targeted to specific cellular compartments such as plasma membrane, nucleus, or mitochondria [20]. As shown in invertebrate *C. elegans*, Prickle can be recruited to the plasma membrane in both a CAAX-dependent and CAAX-independent manner [21]. In vertebrates, however, it remains to be determined whether, and to which content, the CAAX motif with its farnesyl group is important for Prickle localization [22]. The uniqueness of each CAAX sequence (CIIS in both PRICKLE1 and PRICKLE2, CIVA in PRICKLE3, and CTMC in PRICKLE4; **Suppl. Figure 1**) shows that it is not fully conserved, and this suggests their distinct functions within the cell.

Besides PET, LIM, and PKH domains, all Prickle paralogs contain a central intrinsically disordered region, which can be up to 50% of the total protein length (Fig. 1c; **Suppl. Figure 1**). Likely owing to such lengths, the 3D structure of no Prickle paralogs has yet been solved, as intrinsically disordered regions lack a defined 3D structure under physiological conditions. On the other hand, these regions are often associated with PTMs, especially phosphorylation [23]. In line with this, several kinases such as Nemo [24], Misshapen-like kinase 1 (MINK1) [25], and the family of Casein kinases 1 (CK1) [26, 27] have been shown to interact with and modify Prickle. Although these kinases greatly influence Prickle function and localization, it is unknown whether this happens exclusively via the central intrinsically disordered region (see further).

Furthermore, some Prickle paralogs have two unique distinctive elements, VBM and localization signals.

**VBM**. The VBM (Vangl binding motif) is a short and conserved motif that is ~ 100 amino acids long and is unique for Prickle1-2 paralogs. Based on our sequence-based database search, this motif has not been found outside the Prickle family. The VBM binds to the intracellular part of the Vangl protein [10] and is crucial for the proper assembly, localization, and signalling of the PCP complex [10]. This motif was required for Prickle2 asymmetry in *Xenopus* planar-polarized ciliated epithelium, while both LIM domains and VBM promoted Vangl1 asymmetric enrichment [28]. The VBM in PRICKLE2 was also shown to bind to Ankyrin-G, a family of proteins that play a crucial role in maintaining the cell membrane structure, during axonal specification and formation [29]. However, it is unknown whether Ankyrin-G and Vangl compete for the VBM at the same time. As the VBM is not found in both Prickle3 and Prickle4 (Fig. 1c; **Suppl. Figure 1**), it remains elusive which alternative mechanism regulates their asymmetric membrane localization [30].

**Localization signals**. Two localization signals have been predicted for Prickle paralogs. First, mitochondrial localization signal (MLS) was found on the N-terminus of Prickle3 (sequence MFARGSRRRRSGRA in human PRICKLE3) [31], and this sequence is conserved in all vertebrate Prickle3 isoforms (**Suppl. Figure 1**). MLS (or a similar MLS sequence) has not been found in PRICKLE1, 2, and 4 (**Suppl. Figure 1**). Second, several putative nuclear localization signals (NLS) have been predicted for PRICKLE1 and PRICKLE2 with recognition by the importin/karyopherin complex [32, 33]. In PRICKLE1, there are three NLSs at amino acid residues from 617 to 623 (sequence PVLRRSK), 673 to 677 (HRRRR), and 818 to 821 (KKKK; **Suppl. Figure 1**) [32, 33]. As these sequences are conserved in vertebrate Prickle1 and partially in Prickle2 isoforms only, it remains to be determined whether nuclear localization is exclusive to them.

### The similarity in Prickle proteins

To understand the vertebrate Prickle family’s evolution, we performed a phylogenetic analysis. Specifically, we used the UniProt protein database [34] to search for Prickle paralogs from species often serving as vertebrate model organisms like *Homo*, *Pan*, *Mus*, *Xenopus*, and *Danio*. We also added Prickle sequences from invertebrate species such as *Ciona*, *Drosophila*, and *Caenorhabditis* as out-grouping sequences to root a phylogenetic tree. After collecting relevant sequences, we used the MUSCLE algorithm [35] to align the amino acid sequences (**Suppl. Figure 1**) and the Maximum Likelihood method [36] to construct a phylogenetic tree (the detailed procedure is explained in Data availability). Our phylogenetic tree (Fig. 1d) illustrates the evolutionary relationships of Prickle between several species and reveals three pieces of information. First, it classifies vertebrate Prickle proteins into four distinct subfamilies, with Prickle1 and Prickle2 as sister groups, Prickle3 as more distant, and Prickle4 as the most divergent (Fig. 1d). Based on the sequence–structure–function relationship assumption, this suggests that Prickle1 and Prickle2 are more similar not only in sequence, but also in their structure and function properties, and that Prickle3 and Prickle4 are unique family members. Second, the match between the Prickle protein tree and the species tree suggests that the vertebrate Prickle proteins have been subject to a conserved pattern of evolution. Third, the tree showed all individual isoforms are more conserved to each other (e.g. Prickle1 isoforms from all vertebrates are grouped) than to other paralogs among the same species (e.g. human PRICKLE1-4), thus suggesting each isoform has its unique and conserved role within a species over time.

This suggestion is further supported by the sequence conservation analysis. The general assumption behind it is that amino acids crucial for maintaining a protein’s structural or functional properties tend to be conserved over evolution [37]. Although Prickle proteins are highly conserved, this conservancy is reflected on different levels: while the Prickle protein’s amino acid similarity within one species is approximately 20–60% when compared to Prickle1 (Fig. 1e; **Suppl. Figure 2**), the individual Prickle isoforms from different vertebrate species may be up to 80–100% identical, as shown in the example of vertebrate Prickle1 isoforms (Fig. 1e; **Suppl. Figure 2**). On the single amino acid level, the conservation analysis shows that the most conserved residues are found N-terminally, as well as at the C-terminus (Fig. 1f). This conservancy pattern is consistent with the 3D structure formation predicted by the PONDr-Fit tool (Fig. 1g), suggesting that these very conserved amino acids are important for the 3D structure formation of the N- and C-terminus in Prickle proteins. Therefore, this finding emphasizes the importance of the 3D structure formation and amino acid conservation of relevant regions in Prickle proteins.

### Subcellular localization of Prickle

Once *Prickle1-4* are expressed as proteins, they are homogenously distributed throughout the cytoplasm (Fig. 1h) [25, 38–40]. From there, they can be recruited to the plasma membrane by their binding partner Vangl (Fig. 1h), as mentioned previously [5, 6, 10, 25, 28, 38–42]. This membrane distribution, which is crucial for PCP signalling, is induced by PTM such as phosphorylation of the T370 residue in the LIM2 domain in Prickle mediated by MINK1 kinase. Surprisingly, this residue is conserved only in Prickle1 isoforms (**Suppl. Figure 1**) [25], which suggests an alternative mechanism for other Prickle paralogs. While research has proposed that the cytoplasmic form of Prickle primarily acts only as a reservoir for PCP-dependent membrane complex formation, it has also been found to increase F-actin content [43]. In contrast, membrane-bound Prickle reduced the local cortical density of F-actin [43]. These findings suggest that Prickle is essential to determine the actin dynamics for cell rearrangements and migration at the cellular cortex, and this feature very likely depends on PCP signalling.

Although most studies in vertebrates discuss Prickle localization in the membrane in association with PCP signalling, the Prickle1-3 proteins also localize to the nucleus, microtubule organizing centres (MTOCs), and mitochondria (Fig. 1h). First, Prickle1 and Prickle2 were revealed to localize to the nucleus during mouse early development. The presence of Prickle in the nucleus has been shown to be essential for cell fate decisions in the development of the blastocyst cavity, as well as in maintaining the integrity of the trophectoderm during early mouse embryogenesis [33, 44].

As for the first type of MTOC, which is the cilia, Prickle2 was shown to localize to both motile [33, 45] and non-motile cilia such as stereocilia in the inner ear [42]. Sokol and colleagues extended this work by demonstrating that Prickle3 can be involved in ciliogenesis itself, and this might be a PCP-regulated event [46]. As for the second type of MTOC, the centrosome, only the Prickle3 isoform may be involved. Specifically, it was shown to bind a single mature mother centriole and to be delivered to both centrioles during mitosis in ciliated cells. This suggests that the selective interaction of several proteins, including PRICKLE3, to the mature centriole might be necessary for cell polarization and asymmetric distribution of the differently mature centrioles during cell division. However, the root cause of Prickle3 localization to the centrosome and its consequences are not yet fully understood [47–49].

In addition, PRICKLE3 was recently found in mitochondria, where it might be necessary for mitochondrial ATP production and cell bioenergetics [31]. In mitochondria, PRICKLE3 interacted with ATP synthase on the inner membrane by binding to the ATP8 subunit. Cells carrying an R53W mutation in PRICKLE3 (**Suppl. Figure 1**) and a mitochondrial DNA mutation specific for Leber’s hereditary optic neuropathy, the most common maternally inherited eye disease, exhibited defective ATP synthase assembly and stability, leading to ATP synthase deficiency [31]. However, a recent study discovered that cells with only the R53W mutation in PRICKLE3 had a mild decline in mitochondrial ATP contents [50]. Furthermore, they observed little or no increase in cytochrome C, a marker in the apoptotic mitochondrial pathway [50]. These results indicate that the presence of PRICKLE3 in the mitochondria is likely involved in other mitochondrial functions than ATP production and apoptosis.

Based on the similarity and localization data, PRICKLE1 and PRICKLE2 appear to behave in a typical manner for LIM proteins, which involves being present in the membrane (crucial for PCP signalling, Vangl binding, and actin remodelling) and the nucleus (in relation to gene expression and cell fate decision). PRICKLE3, however, is more diverse, as it can be found in mitochondria and MTOCs. Unfortunately, as there is limited knowledge on PRICKLE4, it is difficult to make predictions about its role in vertebrate cells.

## The role of Prickle in vertebrate development

**Fig. 2:**
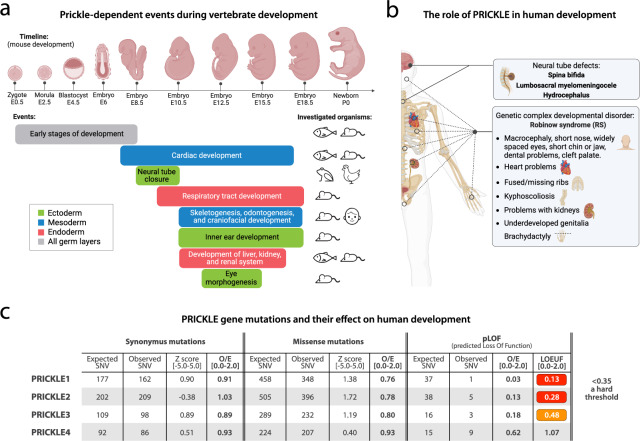
**a** Mapping the Prickle-regulated events during vertebrate development, shown on the example of mouse development. The investigated organisms are depicted on the right. Created with BioRender.com. **b** Developmental defects in humans, regulated by PRICKLE proteins, can be divided into two groups. Created with BioRender.com. **c** gnomAD-derived data showing PRICKLE gene mutations in three categories. Our analysis shows that both *PRICKLE1* and *PRICKLE2* are under the hard pLOF selection, as depicted by low LOEUF values. Higher (more positive) Z scores indicate that the transcript is more intolerant of variation (more constrained). See the text for explanation and abbreviations

The role of Prickle in vertebrate development is complex and multifaceted. Prickle proteins play a crucial role in cell polarization and coordinating key processes during vertebrate development, including early development, neurulation, body axis elongation, and organogenesis (Fig. 2a). Studies conducted in mouse, zebrafish, *Xenopus*, and chicken embryos have revealed the importance of Prickle proteins in these processes. To reflect the presumed role of Prickle in human development, Prickle-regulated developmental defects in humans are discussed at the end of the chapter.

### Early stages of development

During mouse embryo preimplantation development, Prickle1 and Prickle2 were both expressed in the nucleus of 2-cell-stage mouse embryos. If *Prickle1* was deleted, the embryos died between E5.5 and E6.5 [51], which is in stark contrast to surviving *prickle* fly mutants with only a mild phenotype, represented by disrupted bristles on the body surface. For this action in mice, not only Prickle1 expression was required, but also its proper nuclear localization [51]. In the meantime, if *Prickle2* was absent, mouse embryo development was arrested around the 30-cell stage, resulting in blastocyst cavity formation failure and a morula-like appearance [33]. It appears that both Prickle-dependent regulation events occur independently of PCP signalling. First, the deletion of other PCP component mutants such as Dishevelled does not ultimately lead to mouse embryo death [52]. Second, PCP signalling, formerly known as planar tissue polarity or PTP [53], does not occur early for the first time as during gastrulation [54], when a “proper” tissue of cells is formed. Collectively, these findings demonstrate that both Prickle1 and Prickle2 are irreplaceable for proper preimplantation development in vertebrates, and their deletion is lethal for a vertebrate embryo. Thus, vertebrate Prickle1-2 have an additional role compared to its invertebrate homolog originating from, for instance, *Drosophila* and other vertebrate Prickle paralogs, as *Prickle3* [31] and *Prickle4* [55] deletion mouse mutants were viable.

### Neurulation and body axis elongation

Neurulation and body axis elongation are closely related processes that occur during vertebrate development. Neurulation is the ectoderm’s post-gastrulation process which creates the neural tube, and which later gives rise to the brain and spinal cord in vertebrates. Body axis elongation is the mesoderm’s post-gastrulation process during which the body of a vertebrate embryo elongates along its anterior–posterior axis. Both processes require the coordination of multiple molecular components and cellular movements, such as convergent extension (CE). CE is a form of collective cell movement, where cells at the edges of the tissue move towards the centre while simultaneously elongating in the same direction. This is a crucial mechanism for neural tube closure and body axis elongation, as it extends the length of the body axis and reduces the distance between the adjacent neural folds, leading eventually to their fusion [56, 57]. It has been shown that CE is regulated by PCP signalling via directed cell intercalations in the planar plane, and this has been extensively studied in *Xenopus* embryos [6, 56, 58].

**Neurulation**. Endogenous Prickle1 was strongly expressed during embryogenesis in the posterior neural ectoderm, where it persisted through the neurula stage [59], suggesting its involvement in neurulation. During neurulation, Prickle2 colocalized with Vangl2 at the anterior cell edges of the neural plate at NF stage 13 in *Xenopus* development [6], indicating active PCP signalling. This colocalization was enhanced at the shrinking cell–cell junction during cell intercalation of the closing neural tube [60], further supporting its role in neurulation and CE. Recent studies showed that CE is dependent on synchronized oscillatory actomyosin contraction causing the cellular shrinking necessary for cell intercalation [61]. In line with this, Prickle2 was detected to be accumulated at the cell–cell junctions once shrinking starts [60]. Moreover, if *Prickle2* was knocked down by morpholino antisense oligonucleotide (MO) injection, the actomyosin contraction frequency was impaired [61], thus further strengthening the role of Prickle in CE ectodermal processes. In addition to Prickle2, Prickle3 was found to be associated apically with the Par3 protein in the *Xenopus* neural plate and suggested to be involved in CE [62]. As some of these results were shown in a developing chicken embryo with misexpressed Prickle1 [63], these data collectively demonstrate Prickle is involved in PCP-dependent neurulation by CE and this function is likely conserved across different organisms.

**Body axis elongation**. Endogenous Prickle1 was strongly expressed during frog embryogenesis in the dorsal mesoderm too [59], suggesting its involvement in body axis elongation. Indeed, injecting *Prickle1* mRNAs into *Xenopus* embryos’ dorsal blastomeres caused the development of tadpoles with a significantly shorter dorsal axis [10], indicating an issue with the proper mesoderm CE induced by the Prickle1 overexpression. This process is consistent with the overexpression of other PCP components such as Dishevelled [56]. *Prickle1* mutation, on the other hand, impaired cell migration, resulting in cell intercalation failure and subsequent CE defects [43]. To support findings from *Xenopus*, the transplantation of cells with overexpressed Prickle1 to the wild-type environment led to CE defects along the anterior–posterior axis in zebrafish. *Prickle1* MO injections resulted in a shorter body axis, again linking Prickle to mesodermal CE regulation. However, *Prickle2* MO injection showed a less severe phenotype [64, 65], suggesting the privileged role of vertebrate Prickle1 in this process.

Altogether, evidence shows that mostly Prickle1-2 play a critical role in neurulation and body axis elongation by regulating cellular processes such as CE, and their proper balance is essential in these events. As the manipulation of other PCP proteins such as Dishevelled showed similar phenotypes [56], it is clear that the role of Prickle in neurulation and body axis elongation is PCP-dependent.

### Organogenesis

Most research on Prickle-regulated vertebrate organogenesis shown in this subchapter seems to be PCP-dependent, at least based on the studies done predominantly on mouse embryos with mutated *Prickle1* [66–68]. As mentioned earlier, since the mouse null mutant of *Prickle1* is not viable, this early embryonic lethality must be somehow bypassed to further study the role of Prickle in organogenesis. For this purpose, researchers have used the gene-trap technique, which is inducible and allows researchers to control genes temporally [69]. Several *Prickle1* mouse mutant constructs have been created, which we briefly discuss here. The first mutant is Prickle1 C251X (Prickle1^C251X/C251X^) [66], which targets the first cysteine residue C251 in the LIM3 domain (**Suppl. Figure 1**) that forms a zinc finger and changes it to the stop codon (therefore, the whole LIM3 domain, the central disordered region, and the C-terminus of Prickle1 are missing). The second mouse mutant termed *Beetlejuice* (Prickle1^Bj/Bj^) targets C161F [67] (**Suppl. Figure 1**), the cysteine residue in the first LIM1 domain, which forms a zinc finger, and which is conserved in all Prickles including invertebrates (**Suppl. Figure 1**). Finally, another *Prickle1* mutant mouse targets a different exon at the N-terminus, specifically exon 2, and results in the expression of Prickle's very N-terminal only [68]. A reader should consider this information, as it might influence the overall insight into Prickle-regulated organogenesis.

**Skeletogenesis, craniofacial development, and odontogenesis**. Recent studies have shown that PCP signalling plays a key role in various hard tissues’ morphogenesis, including bones and teeth as well as limb elongation and patterning [70–72]. Limb elongation and patterning along the proximal–distal axis are partially mediated by oriented cell divisions and migration, for which the PCP components are crucial [70]. In Prickle1^C251X/C251X^ mutant mouse embryo, the first signs of limb development impairment were described starting from E11.5. This led to the development of shorter limbs at later stages of embryonic development. At E18.5, shorter limbs and impaired vertebrae resulting in a shorter tail were observed. Additionally, these mutants exhibited an increased level of cell death in the digit area and decreased level of apoptosis in interdigit space [66]. Similar phenotypes were also observed in other *Prickle1* mutant mice targeting exon 2, still exhibiting shortened limbs, blunted digits, misaligned sternebrae, and shorter but thicker long limb bones such as scapula, humerus, radius, and ulna [68], thus indicating and supporting the active role of Prickle1 in skeletogenesis.

Congenital defects in the craniofacial region are often manifested by cleft palate. To show Prickle proteins are involved in this process, the Prickle1^C251X/C251X^ mutant embryos exhibited shorter snout and an open palatal shelf [66] and Prickle1^Bj/Bj^ had wider cranial bases than wild-type animals [67]. Furthermore, in the latter mutant model, the cleft lip was observed in all studied foetuses, and cleft palate in 52% of embryos [73]. As the *Prickle1* hypomorph mutant targeting exon 2 exhibited a number of craniofacial defects such as widely spaced eyes, a flat nose, a short snout, and a prominent forehead [68], the role of Prickle1 in proper craniofacial development has been well established.

Evidence of PCP involvement in the odontogenesis process started to appear recently [5, 74]. Expression of Prickle1, Prickle2, Prickle3, and Prickle4 was confirmed in differentiating ameloblasts of rat incisors with Prickle1 and Prickle2 localized specifically in secretory ameloblasts. Prickle3 was predominantly found in the supranuclear cytoplasm of both secretory and mature ameloblasts, and the same applies to Prickle4 [5]. *Prickle1* hypomorph targeting exon 2 exhibited fused mandibular incisors [68], thus collectively suggesting that Prickle1, as well as Prickle2, regulates odontogenesis.

**Eye morphogenesis**. An indispensable step in proper eyelid formation is the elongation of the periocular ectoderm followed by eyelid fold fusion, a process similar to the CE-driven fusion of neural folds (see above). The eyelid fold fusion represents the developmental event for which the PCP pathway represents an important driving force [75]. To test the role of Prickle1 deficiency on eyelid closure, the Prickle1 mutant targeting exon 2 exhibited delayed eyelid closure starting from E15.5. This phenomenon was also accompanied by altered cell orientation and cell shape of the eyelid junctional cells [75]. Furthermore, this mutant showed abnormal morphology of eyelids and eyelashes [68], clearly indicating the role of Prickle1 in mouse eye morphogenesis.

To support the results from mice, Prickle1 expression was present in the retinal ganglion cell layer, inner nuclear layer, and at the lens in 3dpf (days post fertilization) old zebrafish embryos and in the retina of adult fish. A similar expression phenomenon was described for Prickle2 [76, 77], suggesting that not only Prickle1 but also Prickle2 is indispensable for proper eye morphogenesis in zebrafish.

Finally, the role of the Prickle protein family in eye morphogenesis was further supported by the role of mouse Prickle3 in Leber’s hereditary optic neuropathy [31]. However, in contrast to other eye-involved Prickle events, whether this Prickle3 action concerning mitochondria is PCP-dependent or not remains to be proven.

**Inner ear development**. The utricle and saccule of the inner ear are equipped with hair cells that bear bundles of V-shaped actin stereocilia and single tubulin-based kinocilium, both pointing to the abneural edge of the cochlea. Similarly, as in other ciliated organs, the proper function of hair cells is PCP-dependent based on the polarized deposition of kinocilium [78].

As has been demonstrated, asymmetric Prickle2 expression first appeared at E13.5 within differentiating hair cells. Interestingly, it was observed that the Prickle2 expression was present in hair cells with centrally located kinocilium, implying that Prickle2 and PCP signalling is essential to initiate hair cell polarity. On top of that, Prickle2 positive crescents remained until P12, indicating its necessary role not only in initiating hair cell polarity but also in maintaining it [42]. Once the Prickle’s binding partner Vangl2 was mutated in *Vangl2* conditional knock-outs, Prickle2 was mislocalized from the cell boundaries of non-sensory cells situated along the organ of Corti to the random appearance throughout the cell periphery [79]. However, in a *Vangl2* mutant lacking a transmembrane domain, Prickle2 remained preserved in the medial utricle. In contrast, the *Vangl2* ^*Lp/Lp*^ mutant completely lacked Prickle2, resulting in cellular polarity disruption in the same area of the inner ear [80]. Together, this suggests that Vangl2 is an important regulator of Prickle in the inner ear, but the changes in Prickle2 expression differ depending both on the type of *Vangl2* mutation and the observed part of the inner ear.

In the Prickle1^C251X/C251X^ mutant, the cochlear spiral ganglion neurites were not developing properly – neurites grew towards the apex instead of towards the base and failed to protrude and innervate the hair cells. However, the *Prickle1* mutation did not cause any misorientation or PCP impairment of hair cells [81]. In contrast, Liu et al. described shorter and orderless bundles of actin stereocilia in the absence of Prickle1 [68]. To sum up, Prickle1-2 have been found to play an important role in inner ear development, particularly in forming and maintaining hair cell polarity.

**Respiratory tract development**. The respiratory tract is equipped with multiciliated cells, which are necessary for the efficient clearance of respiratory contaminants. Appropriate mucociliary airway cell (MCC) function requires correct cilia orientation along the whole pseudostratified epithelium. For this purpose, PCP proteins represent a key feature for the regulation of proper cilia adjustment [82].

As shown in the cell culture of mouse tracheal epithelial cells, the expression of key PCP proteins was found to be asymmetrical as expected, including Prickle1-4 [83, 84]. In comparison to previous sections where Prickle1 usually dominated, Prickle2 seems to be more relevant here. During embryonic development, as airway epithelial cell differentiation and ciliogenesis proceed, Prickle2 expression initially appeared at E16.5 exclusively in already ciliated cells. Such a delayed and MCC-restricted appearance contrasts with the rest of the PCP core proteins such as Vangl1 and Frizzled6, which are asymmetrically expressed starting from E14.5 across the whole airway epithelium. This implies that Prickle2 is not necessary for intercellular (PCP-like) polarization, but is rather restricted to polarizing cilia in MCCs [83]. When the cilia biogenesis was disrupted, the Prickle2 crescent was missing, suggesting that Prickle2 expression appears to be MCC differentiation dependent. Interestingly, Sowers and colleagues showed that *Prickle2*-deficient adult mice had defective cilia, implying that Prickle2 has an influence on proper cilia formation and function [45]. However, according to Vladar and colleagues, *Prickle2* mutant mice exhibited only limited cilia disruption [30], confirming Prickle 2 as the link between MCC differentiation and PCP protein localization regulation downstream of basal body orientation.

On the contrary to Prickle2, the Prickle1 and Prickle3 isoforms were both expressed in equal amounts in MCCs and other cell types of airway epithelia. The appearance of Prickle4 was similar to Prickle1 restricted to MCCs. As for the latter one, *Prickle1* mutant mice targeting exon 2 revealed a more severe phenotype with a lower number of cilia in the MCC with visible basal body misorientation [84]. This clearly indicates that all Prickle proteins are essential for proper cilia arrangement in the respiratory tract, but Prickle2 can do it in a unique way involving cilia polarization in MCCs.

**Cardiac development**. Cardiac development requires precise formation, septation, and remodelling, and to achieve this, PCP signalling is used to gain proper outflow tract formation. This is evidenced in the *Prickle1* ^*Bj/Bj*^ mutant mice, in which detailed cardiovascular phenotyping uncovered a congenital heart defect in the so-called double outlet right ventricle combined with a perimembranous ventricular septum defect. Furthermore, since PCP signalling is responsible for cardiomyocyte migration, *Prickle1* mutant embryos do not have myocardial prongs and the overall myofilament alignment in the cardiomyocytes is disorganized/misoriented [68, 85].

Studies on zebrafish have also revealed the effects of *Prickle* disruption, as randomized heart looping was observed following Prickle1 and WNT11 elimination by MO injection. This knock-down targeted Kupffer’s vesicle, which is responsible for left–right asymmetry patterning in the brain, heart, and gut [86]. This suggests that Prickle proteins may be involved in Kupffer’s vesicle formation and thus in the left–right patterning of organs like the heart.

**Liver, kidney, and renal system development**. The liver and Prickle proteins are closely connected since PCP is essential for its proper polarization and cell arrangement. In zebrafish, *Prickle1* MO knock-down led to reduced liver biliary size followed by abnormal intrahepatic biliary development. Concurrently, *Prickle1* MO-injected larvae exhibited an increase in abnormal digestive organ localization, such as left-sided liver, gallbladder, intestine, and both exocrine and endocrine pancreas [87]. The biliary duct in *Prickle1* mutant mice was shorter than wild type; however, the missing length was substituted by the increased duct width. Nevertheless, the mutant biliary duct had significantly less mucosal folds and several layers of epithelial cells lining the surface of the liver, that allow performing its various functions [88]. Thus, Prickle1 seems to be indispensable for proper mucosal folding.

The kidneys, as one of the PCP-dependent organs, exhibited cysts at low penetrance and dilated renal tubules once the *Prickle1* is mutated. Similarly, collecting ducts exhibit an irregular elliptical shape and cuboidal epithelial cells of ascending Henle’s loop appeared quadrilateral and pentagon compared to the hexagonal wild-type cells [68]. Thus, *Prickle1* mutation produces kidney developmental defects, all of which are indicative of PCP-dependent organ damage.

In the last decade, evidence has accumulated for the role of the PCP pathway in renal system development [89, 90]. In the ureteric bud of *Prickle1* mutant mice, Vangl2 together with Dishevelled1-3 expression was mislocalized when compared to strictly apical protein expression in wild-type animals. Furthermore, the actin filament distribution was concentrated more laterally [68]. These findings demonstrate the importance of Prickle in the renal system and its role in the proper localization of other PCP proteins and actin filaments.

### Developmental defects in humans

In this chapter, we demonstrated that Prickle is essential for the proper function of various processes during vertebrate development, which involves all three germ layers (Fig. 2a). Its mutations are thus associated with a range of developmental defects in organisms including humans, which can be arbitrarily divided into two groups (Fig. 2b).

The first group is neural tube defects (Fig. 2b), which are among the most common human birth defects with a prevalence between 0.5 and 2 per 1,000 births [91]. Since neural tube closure is a dynamic process, the probability of any mistakes leading to developmental defects represents a real threat. The most common neural tube closure defect, i.e. spina bifida, has been shown to be caused by a mutation in genes belonging to the PRICKLE protein family. Specifically, six *PRICKLE2* single-nucleotide polymorphism variants showed potential association with spina bifida [92]. Another study detected seven rare missense heterozygous mutations in *PRICKLE1* associated with neural tube defects such as hydrocephalus or lumbosacral myelomeningocele. All these mutant variants caused CE perturbation with a large number of severe phenotype observations [93]. Therefore, it is clear that PRICKLE1-2 play a crucial role in closing the neural tube and have the potential to be used in clinical practice for diagnosing neural tube defects.

The second type is Robinow syndrome (RS), a human genetic complex developmental disorder with a prevalence of 1 per 500,000 births. RS involved errors in many PRICKLE-regulated developmental events described above, such as macrocephaly (erroneous neurulation), spine and ribs deformities (erroneous skeletogenesis), cleft palate (erroneous craniofacial development), hearing loss (erroneous ear development), and problems with the heart, kidney, and renal system (Fig. 2b). These RS’ pathological features have been recapitulated and studied in *Prickle1* mutant mice targeting exon 2 [68]. These data demonstrated that *PRICKLE1* deregulation disrupted processes such as cell migration, leading to severe developmental defects like RS.

Developmental defects, in general, are twice as lethal as cancer for people in the Western world, and it is striking how rarely these statistics are discussed [91]. Therefore, we analysed the mutational constraint spectrum quantified from variation in about 140,000 humans, available in the Genome Aggregation Database (gnomAD), a comprehensive database providing information on genetic and functional aspects of human development and the successor to the Exome Aggregation Consortium (ExAC). We checked expected and observed *PRICKLE1-4* single-nucleotide variants (SNV) in three different types of variation: synonymous mutations, missense mutations, and predicted loss of function (pLOF; Fig. 2c). As for synonymous and missense mutations, no major differences between the observed to expected ratio (O/E) were detected. However, the pLOF data regarding O/E and related loss-of-function observed/expected upper bound fraction (LOEUF) values, which enable placing each gene along a continuous spectrum of tolerance to inactivation, showed that only 13% of the expected loss-of-function variants in *PRICKLE1* and 28% in *PRICKLE2* were observed. Therefore, both genes are under hard selection against LOF variants, as 35% is a hard threshold according to the authors [94]. In contrast, *PRICKLE3* and *PRICKLE4* are not under such hard pLOF selection, thus suggesting that they are not so crucial to proper human development. Collectively, these data are in nice agreement with the ones from mouse embryos, where only the deletion of either *Prickle1-2* was shown to be lethal for mice.

## The role of PRICKLE in cancer

Unravelling the complexity of the PCP pathway is essential to gain insights not only into physiological processes but also diseases, particularly cancer progression. A detailed examination of the components of this pathway, such as PRICKLE family proteins, is necessary to comprehend its role in cancer biology and for the development of modern anti-cancer therapies [95, 96]. In this chapter, we discuss the altered expression, mutational profile, and cellular signalling roles of PRICKLE in cancer cells.

### Altered PRICKLE protein expression in cancer

**Fig. 3:**
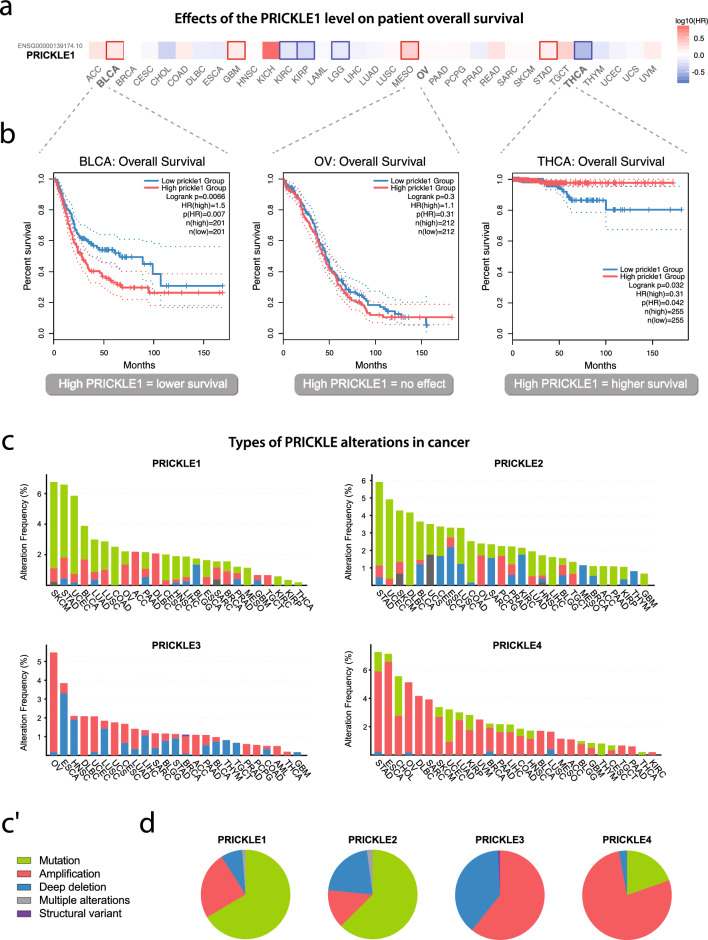
**a** Survival heat map of *PRICKLE1* across the TCGA dataset showing its expression levels. The red blocks indicate higher hazard risk and blue blocks indicate lower hazard risk when *PRICKLE1* expression is elevated. The bold square frame indicates the statistical significance. **b** The diverse impact of the PRICKLE1 high (red) and low (blue) expression level on the overall survival of patients with bladder urothelial carcinoma (BLCA), ovarian serous cystadenocarcinoma (OV), and thyroid carcinoma (THCA). **c** *PRICKLE* isoforms alteration frequencies and types across TCGA cancer studies. Each column represents the indicated cancer type, and the legend (**c’**). The used abbreviations are explained in Supplementary Table 1. **d** Pie charts showing the percentage of alteration types across *PRICKLE* genes

Cancer cells often show extensive alterations in protein expression levels, which are drivers of their malignant transformation [97]. We wondered whether this is the case in PRICKLE proteins. Indeed, PRICKLE has been found to be upregulated in several types of cancers, suggesting it might act as tumour promoting factor. For example, increased expression of PRICKLE1 has been shown to promote cell migration and invasion in breast cancer [98], gastric cancer [99, 100], and leukaemia [101, 102], or PRICKLE4 in the Stem-A molecular subtype of breast cancer [103], together conferring an unfavourable prognosis. However, PRICKLE1 has also been found to have anti-tumour properties in liver cancer [104] and neuroblastoma [105], where its overexpression has been associated with decreased tumour size. Similarly, PRICKLE2 has been reported to have anti-tumour properties in clear-cell renal cell carcinoma [106] and cervical cancer [107], with higher expression levels correlating with longer overall patient survival. Due to the limited number of studies about the role of the PRICKLE isoforms in carcinogenesis, we decided to analyse *PRICKLE1-4* expression in tumour samples compared to normal tissues using the GEPIA2 database (see Data availability). All *PRICKLE* genes had a distinct expression pattern within certain cancer types (**Suppl. Figure 3, 4**), and this heterogeneity was also reflected in the survival data (**Suppl. Figure 5**). In addition, the very same PRICKLE isoform, e.g. *PRICKLE1*, can act as both a negative and favourable overall survival predictor (Fig. 3a, 3b). Based on these data, it seems unlikely that PRICKLE protein levels are *bona fide* drivers of malignant cancer cell transformation. Therefore, we decided to take a closer look at their genes’ mutational profile.

### Mutational profile of *PRICKLE* genes in cancer

It is widely accepted that mutations in genes coding relevant proteins can be the trigger for cancer cell transformation. To answer whether *PRICKLE1-4* might be one of these genes, we analysed the TCGA dataset using the cBioCancer portal (Fig. 3c, 3c**’**). We observed the highest mutational rate of *PRICKLE1* and *PRICKLE2* in melanoma, stomach, and endometrial carcinoma (approximately 6% of all cases). We also noticed that *PRICKLE3* genetic changes were the most common in ovarian and esophageal cancers (5% and 4%), while *PRICKLE4* was affected in more than 6% of the stomach and esophageal tumours. This information suggests that all *PRICKLE* genes are mutated in similar types of cancer with a comparable prevalence of around 5%. However, aberration types varied between genes, with amplifications and deep deletions being predominant in *PRICKLE3* and *PRICKLE4*, and point mutations being the most common in *PRICKLE1* and *PRICKLE2* (Fig. 3d). At the same time, there were no frequent point mutations that we would consider as ‘hot spots’ in tested TCGA cancer cases (**Suppl. Figure 6a**). These data suggest that *PRICKLE* genes are mutated in more than one spot. Finally, we analysed the effect of mutations on patients’ overall survival data, and our analysis revealed that only *PRICKLE3* mutations had a significant negative prognostic impact within TCGA samples **(Suppl. Figure 6b**).

Taken together, these data suggest that mutations are not the most important cause for PRICKLE-regulated tumorigenesis. Thus, PRICKLE should be carefully studied to reveal their individual roles and action mechanisms in cancer biology. To do so, we will discuss PRICKLE proteins’ signalling role in (cancer) cell migration, the most important step of metastasis responsible for 90% of cancer deaths [108].

### Cellular signalling roles of PRICKLE proteins in migrating (cancer) cells

**PRICKLE and cytoskeleton crosstalk**. Cell migration is a complex process that involves cytoskeletal rearrangements and several different pathways, including PCP signalling. Thus, here we aimed to describe known information about the PRICKLE family in cellular locomotion, which might tell us more about its role in cancer. Downregulating PRICKLE1 expression in cancer cells significantly reduced migration speed; however, upregulating Prickle1 expression does not always increase cell migration [109–111]. Daulat and colleagues found that cells with a high basal level of PRICKLE1 were insensitive to further overexpression, and that only cells with a lower initial PRICKLE1 level showed an increase in migration speed [109–111]. These results suggest that the regulation of PRICKLE1 expression is important for controlling cell migration in general, but there is a certain limit for increasing its speed. Furthermore, we asked whether and how PRICKLE localization can influence (cancer) cell migration.

**PRICKLE localisation**. As for cell migration, it can be classified from different views. The two most common categories are amoeboid and mesenchymal, and single and collective [112]. The mesenchymal mode of migration involves changes in the actin cytoskeleton and the formation of various cellular protrusions, including thin extensions of the leading edge called lamellipodia. In single migrating mesenchymal cells, PRICKLE1 was found adjacent to the leading edges together with VANGL2. There, PRICKLE1 was aligned along the non-protrusive membranes that are lateral to the active protrusions. On the contrary, FRIZZLED7 and DISHEVELLED3 were enriched at the tip of migrating cell protrusions [109, 113]. As there is no direct evidence about the PRICKLE protein distribution in different categories of migration, we hypothesize that PRICKLE can be present at the leading edge in collectively migrating cells [114] and at the trailing edge or in the uropod structure in ameboid lymphocytes [115]. Our assumption was made based on the localisation of the VANGL protein in cells, as the asymmetric polarization of PCP components resembles the planar-polarized localisation of the PCP components in epithelial cells in vivo [116] to some extent. The precise mechanism behind the asymmetrical PRICKLE localization in migrating cancer cells is not fully understood, but two things are important to mention. First, it has been suggested that the MINK1 kinase is involved, as it induces PRICKLE membrane localization [25, 111], and MINK1 expression is elevated in chemoresistant carcinomas [117], thus suggesting its possible role in tumorigeneses. Second, the correct subcellular localization of PRICKLE is important for its signalling roles, and this is described below.

**PRICKLE and small GTPases**. Rho and Ras-family GTPases, regulated by the small Rho-guanylyl exchange factors (GEFs) and GTPase-activating proteins (GAPs), are crucial actin cytoskeleton regulators. Spatiotemporal and mutually exclusive interaction between RhoA and Rac1 GTPases control protrusive and retracing motile cell forces, ensuring efficient movement [118, 119]. Strikingly, these signalling events are downstream components of the PCP pathway, and some of the small GTPases are known as PRICKLE1 interactors [25, 109, 120].

**Fig. 4:**
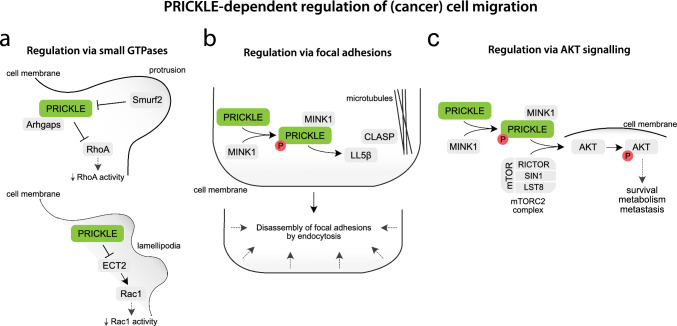
Molecular mechanisms of PRICKLE-dependent signalling regulation in migrating (cancer) cells, via **a** small GTPases, **b** focal adhesions, and **c** AKT signalling

Zhang and colleagues showed that PRICKLE1 and Arhgap21/23 complex, an actin cytoskeleton regulator, together inhibit RhoA activity in the actin-enriched lamellipodia (Fig. 4a) [109]. Similarly, PRICKLE1-depleted cells displayed protrusive membrane ruffling around the entire cell periphery caused by uncontrolled RhoA activity. In cells lacking PRICKLE1, the level of Myosin light chain 2 phosphorylation (pMLC2), the activated component downstream of RhoA, was increased and evenly distributed along the cell membrane, whereas in control cells, pMLC2 was concentrated in the restricted protrusion region [109]. This increase in actomyosin contractility led to impaired cell migration due to PRICKLE1 downregulation [110, 111]. On the other hand, upregulation of PRICKLE1, caused by the silencing of the E3 ubiquitin ligase Smurf2 marking PRICKLE1 for degradation, stopped excessive protrusive activity and decreased the ability of cells to migrate. A precise balance of Prickle1 in lamellipodia is required to control directional cell migration [109, 121, 122]. It is known that overexpression and downregulation of the PCP components can lead to the same phenotypes [123, 124], caused by interference with the dynamics of cytoskeletal rearrangements. Interestingly, increased Prickle1 levels either by overexpression of the exogenous construct [98, 110] or by modulation of the endogenously expressed Prickle1 protein level through the *Smurf2* siRNA [109] produced different effects. It seems that Prickle1 localisation and local concentration control are crucial for the proper regulation of the actin network via small GTPases.

PRICKLE1 was also shown to interact with Epithelial cell transforming sequence 2 (ECT2) in lammelipodia (Fig. 4a) [120]. ECT2 is one of the GEFs that promote Rac1 activity, stimulating cell growth and invasion [125]. Upregulating PRICKLE1 inhibited ECT-induced Rac1 activation, suggesting that PRICKLE1 is a negative ECT2 regulator [120]. Evidence suggests that PRICKLE1 contributes to the Arhgap21/23 and ECT2 spatial localisation in order to modulate RhoA and Rac1 activity, which is vital for reorganizing the actomyosin network and subsequent cell migration [126].

**PRICKLE and focal adhesions**. Focal adhesions are protein complexes facilitating the interaction of cells with the underlying extracellular matrix. PRICKLE1 was also found to be in close proximity to those structures, and its depletion has been shown to lead to the formation of large, stable focal adhesions and impaired cell migration (Fig. 4b). The knock-down of either PRICKLE1 or MINK1 kinase increased the level of active β1-integrin, a protein involved in focal adhesion structure and maturation [111, 127].

Furthermore, PRICKLE1 has been found to interact with two proteins involved in focal adhesion turnover: CLIP-associating proteins (CLASPs, two isoforms CLASP1 and CLASP2) and Pleckstrin Homology Like Domain Family B Member 2 (PHLDB2, also known as LL5β) [110, 128]. CLASPs promote the stability of microtubules and anchor them to focal adhesions, allowing for their disassembly [129]. LL5β, on the other hand, is responsible for recruiting CLASPS to the plasma membrane [130]. PRICKLE1 downregulation did not affect LL5β localisation in the distal regions of the cell cortex, but abolished CLASP1 recruitment to this site. Moreover, LL5β knock-down impaired PRICKLE1 localisation in focal adhesions [110], suggesting that PRICKLE1 is upstream from CLASP1 and downstream from LL5β. Additionally, LL5β is also known as a substrate for MINK1. MINK1 phosphorylates LL5β within the CLASPs’ binding domain, which enhances the association between CLASP2 and LL5β at the cell cortex [128]. Thus, PRICKLE1, MINK1, and the CLASP–LL5β complex form a network of interactors that regulate cell communication with the microenvironment by modulating focal adhesion dynamics and cell migration speed.

**PRICKLE and PI3K/AKT/mTOR signalling**. PI3K/AKT/mTOR signalling is conserved and controls various aspects of cell biology. PRICKLE1 was shown to participate in the regulation of the mammalian target of the rapamycin (mTOR) signalling pathway in cancer (Fig. 4c) [98, 100]. It is not clear which mTOR signalling branch is involved in PRICKLE1-dependent cancer cell motility. Zhuo and colleagues showed that inhibiting mTOR signalling by rapamycin, a known inhibitor of the mammalian target of rapamycin complex 1 (mTORC1), decreased migration of the PRICKLE1 overexpressing cells [100, 131]. On the other hand, Daulat and colleagues found that PRICKLE1 interacted only with mTORC2 called RICTOR, and not with RAPTOR involved in mTORC1 [98]. The interaction between PRICKLE1 and RICTOR was positively regulated by MINK1. MINK1 downregulation led to the delocalisation of both proteins from the cell cortex. Similarly to PRICKLE1 and MINK1, downregulating RICTOR influenced cytoskeleton reorganization, increased focal adhesion size, and consequently decreased cell migration [98]. These results are in line with previous findings showing that the mTORC2 complex participates in cytoskeletal reorganization by regulating Rho GTPase activity [132-134] and regulates focal adhesion dynamics [135].

Furthermore, AKT kinase is a known substrate for mTORC2, specifically activated by S473 phosphorylation [136, 137]. Upregulating PRICKLE1 promotes AKT phosphorylation at S473; however, it depends on its interaction with MINK1 and mTORC2 [98]. This is interesting and worth further attention, because the phosphoinositide 3-kinase (PI3K)/AKT signalling pathway plays an enormous role in various types of cancer, regulating cell survival, metabolism, and metastasis [138]. Moreover, it dictates the asymmetric localisation of the noncanonical WNT receptor FRIZZLED6 [139], and its downregulation causes neural tube defects [140]. Thus, the MINK1-PRICKLE1-mTORC2 complex may serve as a local AKT activation unit and promote cytoskeleton reorganization, cell motility, and proliferation.

Overall, what makes PRICKLE unique in cancer is not its protein level or mutational profile, but its cellular signalling roles and their balance. Thus, PRICKLE can act both as a tumour-promoting and as a suppressing factor, since it is involved in miscellaneous downstream pathways. Therefore, we suggest one should investigate the role of PRICKLE proteins in (cancer) cell biology carefully.

## The role of PRICKLE in non-cancer pathologies

**Fig. 5:**
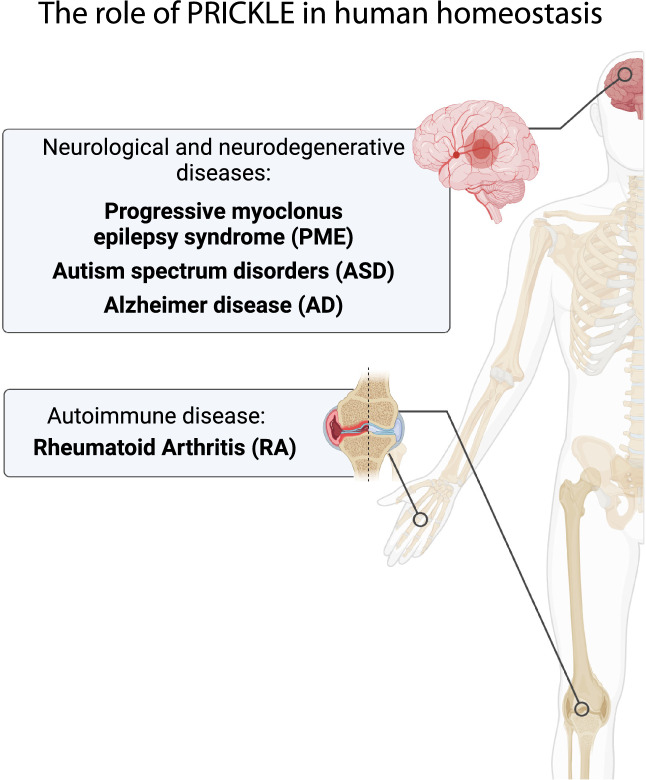
Mapping of PRICKLE-regulated pathologies during the adult homeostasis in humans. Created with BioRender.com

In addition to contributing to tumorigenesis, PRICKLE paralogs have been associated with several neurological and neurodegenerative diseases, including Progressive myoclonus epilepsy syndrome, Autism spectrum disorders, and Alzheimer’s disease, and the autoimmune and inflammatory illness Rheumatoid Arthritis (see Fig. 5).

**Progressive myoclonus epilepsy syndrome (PME)**. Multiple studies have demonstrated various *PRICKLE1-2* mutations linked to autosomal recessive and autosomal dominant PME, a brain disorder characterized mainly by myoclonic and tonic–clonic seizures, balance problems, and neurological decline, especially ataxia and dementia [141].

One of the first studies described a single, missense R104Q mutation in PRICKLE1 (**Suppl. Figure 1**) causing autosomal recessive PME. This mutation was located in the PET domain, disrupting the PRICKLE1 interaction with the crucial neural gene regulator RE1-Silencing Transcription Factor (REST). This, in turn, prevented the regular REST transport out of the nucleus, leading to the constitutively active REST deregulating its target genes and silencing neuronal genes in neuronal precursors, mature neurons, and non-neuronal cells. In patients, it manifested as PME-ataxia syndrome [142]. Moreover, Algahanti and colleagues have recently described a novel autosomal dominant mutation of *PRICKLE1* (R84N; **Suppl. Figure 1**), also affecting the PET domain. Interestingly, this mutation has been also detected in PME-unaffected heterozygous individuals, suggesting that such *PRICKLE1* mutations have incomplete penetrance [143]. These results point to the PRICKLE1-REST interaction’s essential role in the proper function of neuronal cells.

In addition to PRICKLE1, PRICKLE2 has also been associated with PME. Two more heterozygous missense mutations in *PRICKLE1* (R144H located in the LIM1 domain and Y472H in the central disordered region; **Suppl. Figure 1**) and three missense mutations in *PRICKLE2* (R148H and V153I located in the LIM1 domain, and V605F in the central disordered region; **Suppl. Figure 1**) were identified in other patients with PME [144]. The expression of *prickle* zebrafish mutants altered in these amino acids showed aberrant Prickle function and reduced Ca^2+^ activation, indicating that Prickle also mediates Ca^2+^ signalling in the nervous system [144]. We speculate that this could be another molecular mechanism behind PRICKLE-mediated PME.

As a third possible mechanism behind PRICKLE-mediated PME, we suggest PTM PRICKLE de-ubiquitination. Paemka and colleagues identified the de-ubiquitinase USP9X, which is the PRICKLE2 stabilizer in the neural system. Specifically, they showed that USP9X deficiency led to the downregulation of the Prickle2 protein level in the forebrain neurons of mice. Moreover, they identified several patients suffering PME-carrying mutations in USPX9. The authors tested the small-molecule USP9X inhibitor Degrasyn/WP1130, which resulted in decreased PME phenotype in mutant flies [145]. However, these results need to be replicated in human cells. Thus, USPX9 has emerged as a new potential target to treat USPX9 (and perhaps also PRICKLE-) -mediated PME.

**Autism spectrum disorders (ASD)**. Another group of neurological disabilities linked to *PRICKLE* gene mutations is ASD. ASD is a term used to describe a range of conditions that affect social interaction, communication skills, and behaviour. Sowers and colleagues identified two families with ASD whose members carried two missense mutations in *PRICKLE2* located at the very N-terminus (E8Q) and in the LIM1 domain (V153I; **Suppl. Figure 1**) [144, 146]. The function assays showed that disrupting Prickle2 in these amino acids decreased the number of synapses in hippocampal neurons and reduced post-synaptic density size in mutant mice. Loss of *Prickle2* also led to decreased basal synaptic transmission and reduced the number and size of miniature synaptic currents. Moreover, they showed that ASD-like symptoms are already present in heterozygous mice, suggesting that *Prickle2* haploinsufficiency is enough to cause ASD in patients [144, 146]. Besides missense mutations, PRICKLE2 was identified as the most likely cause of ASD-like behaviour in monozygotic twins carrying de novo 3p14 6.88-Mb deletions containing 17 genes involving *PRICKLE2* [147]. These data confirm that one of ASD’s causes is PRICKLE2-mediated synaptic dysfunction.

In addition to PRICKLE2, PRICKLE1 was also revealed to be involved in ASD [148-150]. The study showed that the loss of the *Prickle1* allele led to ASD-like phenotype in mice, including abnormal circadian rhythm and abnormal social and repetitive behaviours. Moreover, Prickle1 has emerged to interact with the synaptic protein Synapsin1 in the mouse brain, which participates in synaptogenesis, synaptic vesicle trafficking, and regulating neurotransmitter release. Mutations in both SYNAPSIN1 and PRICKLE1 led to defects in vesicle pool size and trafficking [148, 151], suggesting that they cooperate to ensure synapse function often impaired in ASD. Another approach consisting of analysing transcriptome organization between autistic and normal brains showed significant *PRICKLE1* expression differences between the frontal and temporal cortex in control and autism samples [150]. Together, these data confirm PRICKLE1’s crucial role in synapse function and ASD.

Because ASD is a neurodevelopmental disorder and patients typically display symptoms before the age of three, one of the key questions in autism research is whether the pathology is reversible in childhood, juvenile, or adult ages. Studies in several models have addressed this issue in genetic animal models (discussed elsewhere [152, 153]), but it remains to be determined whether PRICKLE can be also used for such therapeutic purposes.

**Alzheimer’s disease (AD)**. PRICKLE2 has also been revealed to participate in the most common progressive cognitive neurodegenerative disease, AD. A recent study has shown that APP/PS1/Tau transgenic-AD mice (homozygous for the *Psen1* mutation, homozygous for the co-injected *APPSwe* and *tauP301L* transgenes) display significantly lower *Prickle2* mRNA levels in the brain’s cortex and hippocampus [154]. On the other hand, upregulating the *Prickle2* mRNA levels led to improved cognitive deficits and AD-like pathology. Moreover, the data proved that Prickle2 inhibits the PCP signalling pathway in AD [154]. These results demonstrated that Prickle2 has an essential role in AD and was revealed as a potentially valuable candidate for AD diagnosis and treatment.

**Rheumatoid Arthritis (RA)**. *PRICKLE1* deregulation has been shown to be a possible cause of an autoimmune and inflammatory disease, RA. RA is characterized by the “tumour-like” behaviours of fibroblast-like synoviocytes (FLS), including abnormal proliferation, migration, and invasion [155]. The recent study by Yang and colleagues showed that PRICKLE1 plays an essential role in activating the mTORC2 signalling during irregular FLS cell migration. The authors showed that flavonoid Morin prevents FLS migration and reduces focal adhesion turnover in arthritic rats by targeting a Prickle1-specific stabilizer, ubiquitin-specific protease 7, suggesting that Prickle1 PTM ubiquitination plays a role in RA development [156].

In summary, PRICKLE is involved in pathologies that are mostly connected with the neural and immune system in humans. This is in line with its role in vertebrate development and cancer that we described in the previous chapter, as Prickle plays a crucial role in neurulation and cancer cell migration, which in some features resembles RA.

## Concluding remarks

Prickle proteins are essential PCP mechanism components, with their conservation across the entire animal kingdom indicating their crucial role in vertebrate organisms’ development and pathogeny. Prickle is a relatively new protein and its role in cell development and organization is still being studied. That is why we assume Prickle has not been properly reviewed so far, as its function is still not completely understood.

In the first part of the review, we explored the sequence and structure properties of vertebrate Prickle proteins. We conducted a phylogenetic analysis showing conservation among all vertebrates. We showed that Prickle1 and Prickle2 are more similar to each other, and this finding has been supported many times also functionally throughout the review. Furthermore, we discussed Prickle proteins’ key sequence elements, such as the PET, LIM, and PKH domains, as well as several motifs, which are unique and highly conserved for individual isoforms across vertebrates. This conservancy indicates that each isoform has its defined roles in cellular signalling. At the end of the first chapter, we analysed Prickle proteins’ amino acid conservation and the importance of their intrinsically disordered regions, together with their subcellular localization. The Prickle protein family localizes to the various subcellular compartments in eukaryotic cells such as the cytoplasm, plasma membrane, MTOCs, mitochondria, and nucleus. It seems that this is due to several reasons. In the cytoplasm, Prickle is involved in signal transduction and metabolic pathways. In the membrane, Prickle is involved in the PCP-mediated cell–cell communication. In the nucleus, Prickle may be involved in gene expression and other nuclear processes, and this is important for the viability of vertebrate embryos. In the mitochondria, Prickle may be involved in energy metabolism and other mitochondrial functions. Finally, in the MTOCs, Prickle may be involved in ciliogenesis, cell division, and other centrosomal processes. We speculate that each Prickle protein is likely suited for its specific task, localizing to the compartment where it is needed most and that these processes are regulated by PTM.

In the second part of the review, we explored vertebrates’ embryonic development. It is evident that Prickle plays a critical role in many vertebrate developmental events. In particularly, Prickle1-2 proteins are essential for normal preimplantation development in vertebrate embryos, as their deletion leads to embryonal lethality in mice (based on experimental data) and humans (gnomAD database). All Prickle proteins play a crucial role in neurulation and proper organogenesis. From the obtained data, it seems that some of the Prickle proteins may likely be functionally redundant here. The involvement of Prickle proteins in these processes is conserved across species, emphasizing their importance in vertebrate development, especially in humans, as several developmental defects have been observed and studied.

In the third part, we focused on PRICKLE-regulated pathogeneses. We discussed that PRICKLE proteins are involved in cancer biology. It appears that the function of PRICKLE proteins in the context of cancer is highly context-dependent, and the roles of individual PRICKLE isoforms require further investigation. Moreover, PRICKLE proteins appear to interact with several other important proteins involved in cytoskeletal reorganization and metabolism in cancer cells, suggesting that they play a crucial role in tumorigenesis and metastasis. Of note, the data support the hypothesis that Prickle’s localization and signalling is just as important as its expression. Specifically, PRICKLE expression is necessary to establish PCP, while localizing and signalling Prickle is necessary to maintain PCP. Without proper PRICKLE localization and signalling, the PCP pattern will not be maintained over time.

Finally, we showed that PRICKLE expression and function have been associated with various non-cancer pathologies. Their role in various neurological, neurodegenerative, and autoimmune diseases has been studied in recent years, with many promising findings. PRICKLE proteins’ regulation and dysregulation provide valuable insight into the development and progression of diseases and potential therapeutic targets to diagnose and treat Progressive myoclonus epilepsy syndrome, Autism spectrum disorders, Alzheimer’s disease, and Rheumatoid Arthritis.

## Future perspectives

Going forward, it is essential to explore if Prickle activity is always reliant on Vangl and consequently PCP signalling. A bit of research has been done in this regard already. Notably, the nuclear activity of Prickle1-2 seems to be independent of classical PCP, as PCP occurs later during gastrulation and on. In adult organisms, one such scenario is the presence of PRICKLE in proximity to the focal adhesions, which was not affected by the knock-down of other PCP and PCP-related components such as WNT5 ligands, DISHEVELLED and VANGL proteins, nor by the inhibition of WNT ligand processing and secretion [110]. This suggests that PRICKLE localisation in focal adhesions could be independent of both canonical WNT signalling and PCP pathways. With more research, we could finally provide an answer to this question.

Furthermore, Prickle protein investigations should also include the study of the two underappreciated members of the vertebrate Prickle family: Prickle3 and Prickle4. This is of great importance to understanding their functions and roles in the development and progression of different pathologies. The redundancy of the Prickle family in many cell types is not known. Do Prickle1-4 and their expression levels have a distinct effect on cell signalling? Therefore, further insights into the exact role of Prickle1-4 in cell biology are needed, as current studies primarily focus on Prickle1 and Prickle2.

Subsequently, our phylogenetic tree showed that surprisingly, vertebrate Prickle4 proteins were more closely related to invertebrate Prickles than the other isoforms. As there is little knowledge available about Prickle4, it is not straightforward to understand what this observation implies. Yet, it appears to suggest that Prickle from invertebrates is similar to Prickle4, whereas Prickle1, 2, and 3 have been duplicated and adapted for vertebrate (i.e. more complex) PCP signalling. It would be interesting to explore this hypothesis further, to see if there is any evidence that suggests Prickle4 is indeed an ancestral Prickle protein. To support this hypothesis, the deletion of *Prickle4* in mice does not lead to embryonic lethality, similar to the deletion of invertebrate *Prickle* in *Drosophila*.

In addition, the conservation analysis highlighted the need to validate some described findings. For example, one may notice the importance of conserved residue T370 and its role in Prickle function via MINK1 kinase. However, T370 is conserved only in Prickle1 isoforms, so how is this residue alternated in other isoforms? Similarly, the Prickle part binding to Vangl called VBM is conserved only in Prickle1 and 2, so more research should be conducted to understand how Prickle3 and 4 are performing these functions.

Finally, we would like to point out that induced pluripotent stem cell (iPSC) technology provides a promising approach to better understand *PRICKLE* mutant-related diseases such as ASD [157]. The ability of iPSCs to generate a variety of brain cells combined with the formation of 3D organoids makes them an ideal model for elucidating disease mechanisms [158]. Gene editing tools such as CRISPR/Cas9 have further enabled the study of these mechanisms by allowing specific gene mutations’ control and manipulation [159]. These technologies also promise to eventually create potential therapeutic interventions for these diseases. While in vitro models are important in studying *PRICKLE* mutation-related diseases, animal models also play a valuable role to assess disease pathologies, especially in the context of ASD [160]. Animal models enable further manipulation of specific gene mutations to better understand their interaction with ASD and they also provide opportunities to investigate drug therapies and develop clinically relevant symptomatic treatments for ASD [160]. To sum up, iPSCs and animal models can become essential tools for studying *PRICKLE* mutant-related diseases like ASD and ultimately develop effective therapeutic interventions in the future that can significantly improve the quality of life of all those affected.

In conclusion, the current review provided an overview of the Prickle proteins, their functions, and their roles in different pathogeneses. However, there is still much to uncover and understand, such as the discovery of new PRICKLE-related functions, the exploration of the Prickle family’s evolutionary history, and the validation of already known functions. Thus, this review serves as a valuable tool for researchers looking to unravel the PCP signalling’s implications in animal development and homeostasis. We hope that this review of Prickle proteins will be useful for future studies exploring their structure, function, and evolution and will provide a platform for novel discoveries in the field of PCP signalling.

### Supplementary Information

Below is the link to the electronic supplementary material.

Supplementary file1 (PDF 6014 KB)

## Abbreviations

AD: Alzheimer's disease
ASD: Autism spectrum disorders
CE: Convergent extension
CK: Casein kinase
CLASP: CLIP-associating protein
ECT: Epithelial cell transforming sequence
EMT: Epithelial–mesenchymal transition
ExAC: Exome aggregation consortium
FGF: Fibroblast growth factor
FLS: Fibroblast-like synoviocytes
GAP: GTPase-activating protein
GEF: Rho-guanylyl exchange factor
gnomAD: Genome aggregation database
iPSCs: Induced pluripotent stem cells
LIM: Linl-1, Isl-1, Mec-3
LHON: Leber’s hereditary optic neuropathy
LMO: LIM domain only
LOF: Loss of function
LOEUF: Loss-of-function observed/expected upper bound fraction
MCC: Multiciliated cells
MINK: Misshapen-like kinase
MLS: Mitochondria localization signal
mTOR: Mammalian target of the rapamycin
MTOC: Microtubule organizing centre
MO: Morpholino antisense oligonucleotides
NLS: Nuclear localization signal
O/E: Observed to expected ratio
OEBT: Overexpressed breast tumour
PCP: Planar cell polarity
PET: Prickle, espinas, testin
PHLDB: Pleckstrin homology like domain family B member
PI3K: Phosphoinositide 3-kinase
Pk/PK: Prickle/PRICKLE
PKH: Prickle homology
pLOF: Predicted loss of function
PME: Progressive myoclonus epilepsy
pMLC: Phosphorylation of myosin light chain
PTM: Posttranslational modification
RA: Rheumatoid arthritis
REST: RE1-silencing transcription
RS: Robinow syndrome
SNV: Single-nucleotide variant
VBM: Vangl-binding motif

## Declarations

### Availability of data and material

For Fig. 1d, sequences of 23 Prickle paralogs were collected in the Uniprot database: Q96MT3, Q7Z3G6, O43900, and Q2TBC4 for human PRICKLE1-4; A0A2I3TH70, A0A2I3RJX6, K7DBC5, and H2R2U9 for *Pan* Prickle1-4; Q3U5C7, Q80Y24, Q80VL3, and D3Z6Q6 for mouse Prickle1-4; Q90Z06, A0A8J0V3T3, A8WH69, and D3Z6Q6 for *Xenopus laevis* Prickle1-4; A0A6I8QKU1, F6U3C9, A0A803K7N2, and B0JZ01 for *Xenopus tropicalis* Prickle1-4; and Q6WGK5, Q7ZZC4, and F1Q568 for *Danio rerio* Prickle1-3. Multiple sequence alignments were performed using the ClustalW algorithm, implemented in the BioEdit editor 7.2 [161]. Prickle phylogenetic tree was generated from a set of 23 Prickle sequences above plus Q9NDQ9 for *Ciona intestinalis* Prickle, A1Z6W3 for *Drosophila melanogaster* Prickle, and A0A260ZPH8 for *Caenorhabditis elegans*. Specifically, the sequences were first aligned using ClustalW algorithm, implemented in BioEdit editor 7.2 [161]. The total sum of amino acids and gaps (i.e. regions of insertion/deletion) in the multiple sequence alignment was 1170, and has been visualized in JalView2 desktop version with standardized Clustal X Colour Scheme (Suppl. Figure 1). Next, the best fitting amino acid substitution model and evolution parameters of evolution were estimated using SMS: Smart Model Selection in PhyML [162], with Akaike information criterion (available online on http://www.atgc-montpellier.fr/phyml/). The JTT model [163] was estimated as the best fitting model, in variant JTT + G + I + F (parameter gamma = 1.032, proportion of invariable sites estimated 0.043, and number of substitution rate categories 4). Based on the multiple sequence alignment and JTT substitution model, the (unrooted) phylogenetic tree was calculated using PhyML3.0 [164]. The *Ciona*, *Caenorhabditis* and *Drosophila* Prickle sequences were used here as an outgroup for a tree rooting. Finally, the phylogenetic tree was visualized using PRESTO (a Phylogenetic tReE viSualisaTiOn; implemented in PhyML 3.0 [164]), and manually edited in the Affinity Designer software.

In Fig. 1e, 1f and Suppl. Figure 2, aligned sequences together with their conservation score (see below) were manually graphically edited in the Affinity Designer software. Conservation is visualized on the alignment or a sequence group as a histogram giving the score for each column. Pairwise Sequence alignment and Conservancy score were calculated via Emboss needle tool (https://www.ebi.ac.uk/Tools/psa/emboss_needle/) and the Jalview2 desktop version, respectively.

Figure 1g was created using PondrFit tool available on http://original.disprot.org/pondr-fit.php, and manually edited in the Affinity Designer software.

Figures 2a, 2b, and 5 were created with BioRender.com, the online app used to create, edit, and collaborate on scientific diagrams, and illustrations. Data for Fig. 2C were adopted from the gnomAD database v2.1.1, available on https://gnomad.broadinstitute.org on February 10, 2023.

Data for Fig. 3, including Suppl. Figures 3, 4, 5, and 6 were prepared using GEPIA2 and cBioPortal databases, and manually edited in the Affinity Designer software.

GEPIA2 database analysis: the datasets analysed for this study can be accessed through the following website: http://gepia2.cancer-pku.cn. TCGA and GTEx data were accessed on January 9, 2023. The gene expression profile and box plot function were used to compare cancer versus normal *PRICKLE1-4* tissue expression, by comparing cancer TCGA with normal TCGA and GTEx data. The expression data were log2 (TPM + 1) transformed, with the log2FC = 1 cut-off and q-value cut-off = 0.01. Statistical significance between cancer and normal samples was analysed by ANOVA. The impact of *PRICKLE1-4* expression on Overall survival of TCGA cancer patients was visualized with the help of the survival heat map, using the median for cut-off, with no p-value adjustment. Log-rank significance level was p = 0.05, estimated using the Mantel–Cox test. For overall survival (OS) representation, Kaplan–Meier curves were generated, and the median was selected as the threshold for separating the high- and low-expression groups in TCGA data. The hazard ratio (HR) was calculated based on the Cox PH model; a 95% Confidence Interval was added (dotted line). Graphs were manually edited in Affinity Designer.cBioPortal analysis: The datasets used for this study can be accessed through the following website: https://www.cbioportal.org. TCGA data were used for further analysis, accessed on January 9, 2023, and analysed using software-derived tools. Pie charts representing derived mutational data were generated with the help of the GraphPad Prism 8.0 software. Graphs were manually edited in the Affinity Designer software.

All figures in this research paper were designed with colourblind-friendly colours to ensure maximum accessibility.
