# Supplementary material for: The role of prickle proteins in vertebrate development and pathology
Source: Mol Cell Biochem. 2023 Jun 26;479(5):1199–221. doi: 10.1007/s11010-023-04787-z (PMC11116189; doi:10.1007/s11010-023-04787-z)
Supplement: Supplementary file 1 — Supplementary file1 (PDF 6014 KB) [file 11010_2023_4787_MOESM1_ESM.pdf]

Supplementary Information (SI)

SI contains 6 Supplementary Figures, 1 Supplementary Tables, and the Legend

Supplementary Figure 1

Suppl. Fig. 1 is provided on page 2.

Supplementary Figure 1. The multiple sequence alignment of 26 Prickle sequences. All the sequence elements including MLS, NLS, and the amino acid residues used in the text are highlighted. For more info about the procedure, go to the Data availability section.

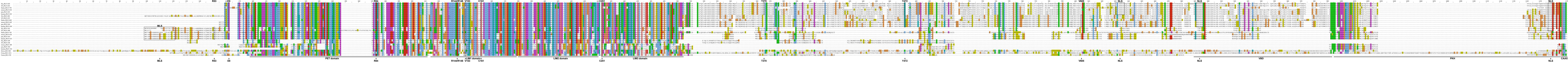
NLS
R53
E8
PET domain
R84
R144/R148
V153
LIM1 domain
C161
LIM2 domain
C251
LIM3 domain
T370
Y472
V605
NLS
NLS
VBD
PKH
NLS
CAAX

Supplementary Figure 2

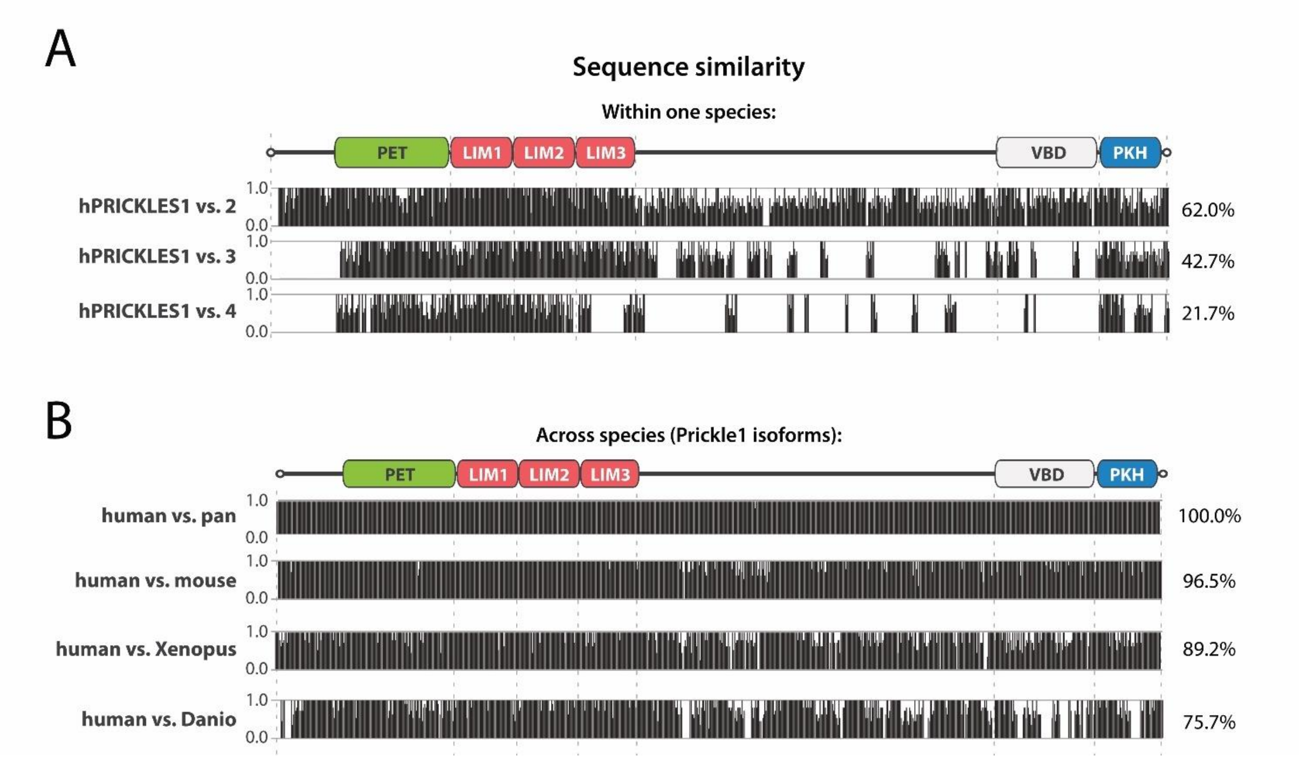

Supplementary Figure 2. a) Amino acid sequence conservation and similarity in individual human PRICKLE1-4, and **b)** Prickle1 isoforms across vertebrates. For more info about the procedure, go to the Data availability section.

Supplementary Figure 3

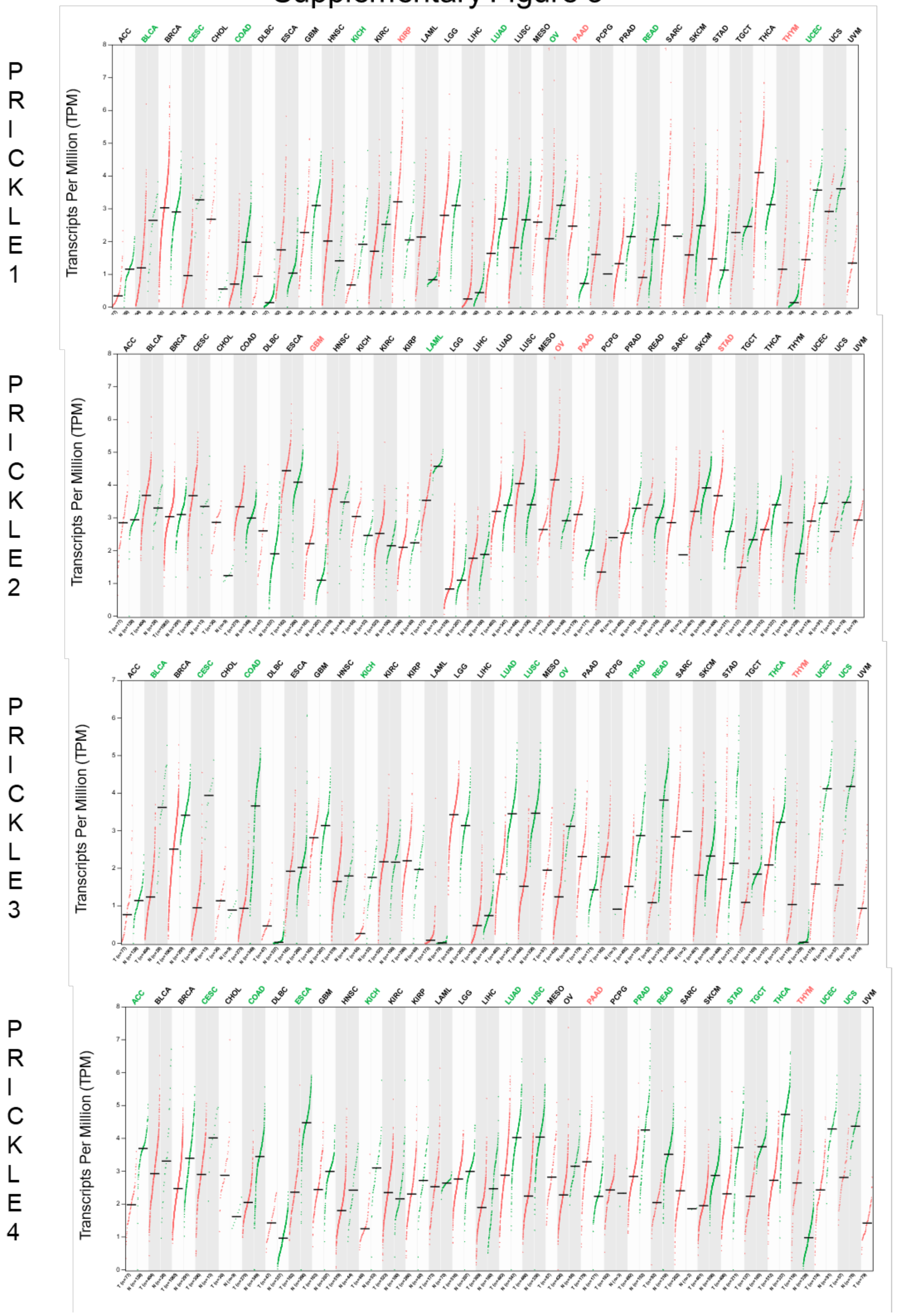

Supplementary Figure 3. *PRICKLE1-4* expression across cancer types in comparison with corresponding normal tissues. The column graph displays normal samples (N) as green and tumour (T) as red dots. Cohort size is shown (n). TCGA study abbreviations encoding cancers with significantly upregulated PRICKLE isoforms are highlighted in red and downregulated in green.

Supplementary Figure 4

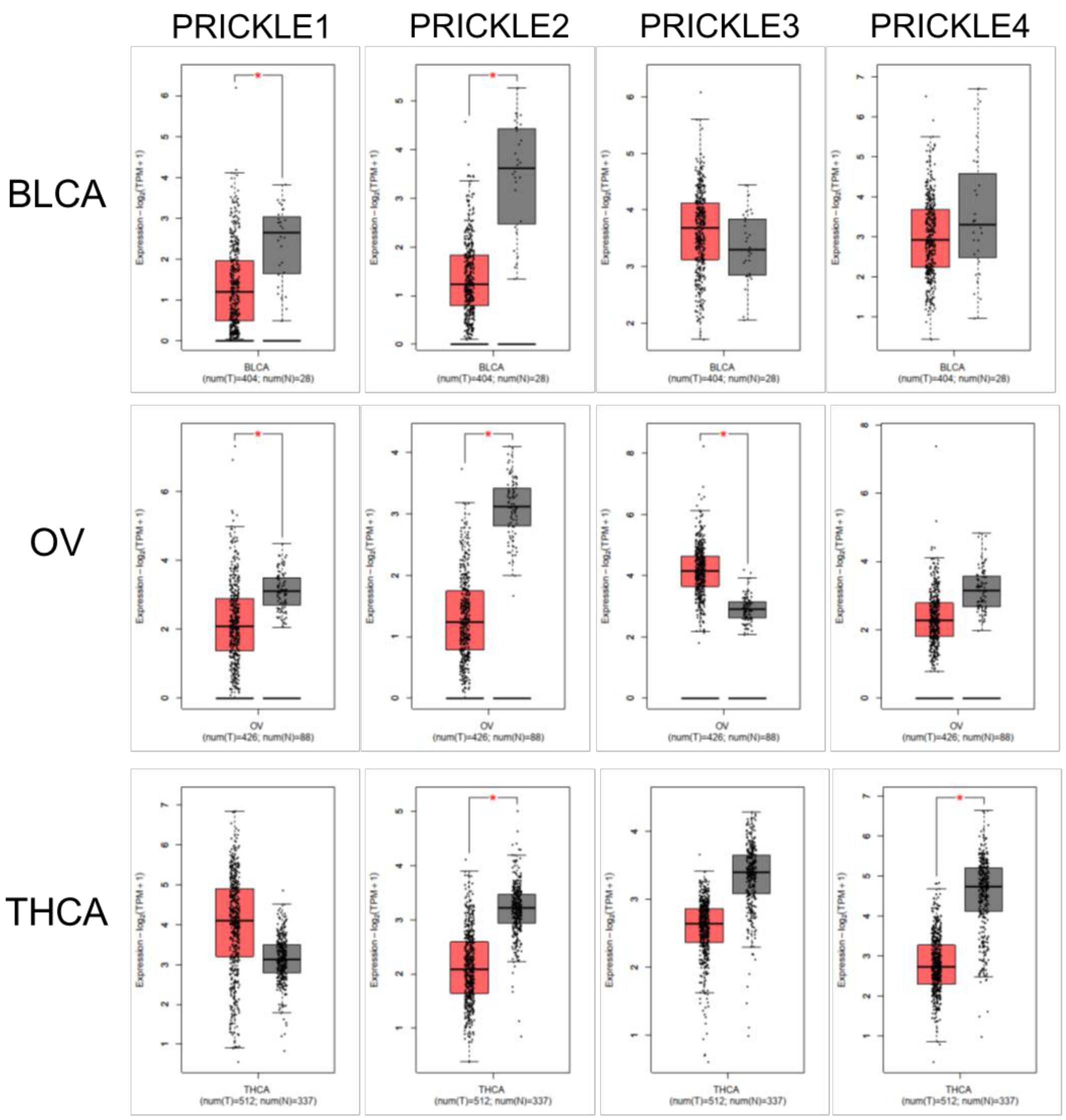

Supplementary Figure 4. Box plots – expression levels of *PRICKLE* isoforms in patients with Bladder Urothelial Carcinoma (BLCA), Ovarian serous cystadenocarcinoma (OV), and Thyroid carcinoma (THCA) were investigated; Tumour samples (T) represent a red box; health tissue a grey one.

TPM – transcripts per million in the selected TCGA cancer types.

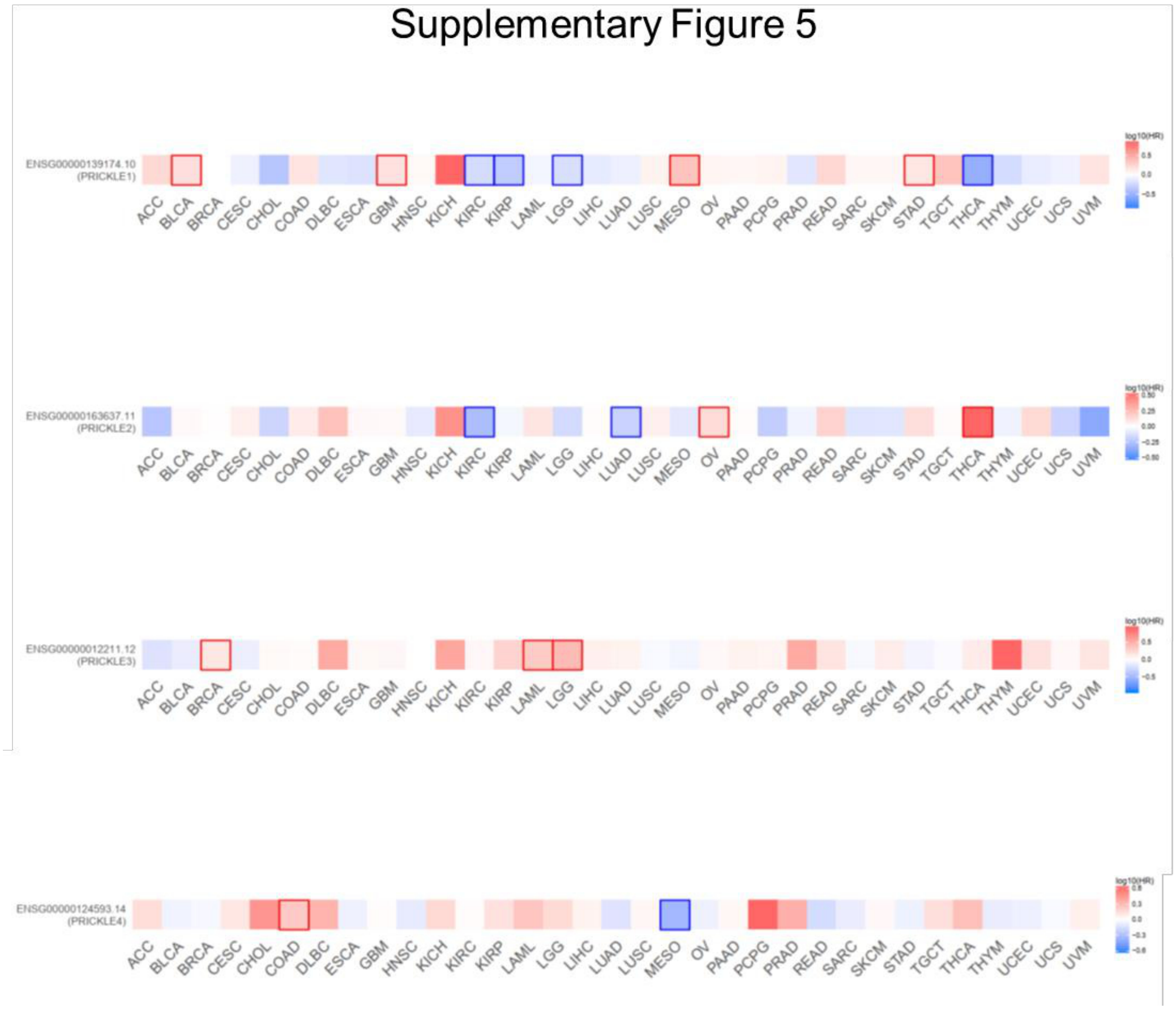

Supplementary Figure 5. Survival heat map of *PRICKLE1-4* across the TCGA dataset. The red blocks represent higher and blue blocks lower hazard risk, when gene expression is altered. Bold square frames indicate the statistical significance.

Supplementary Figure 6

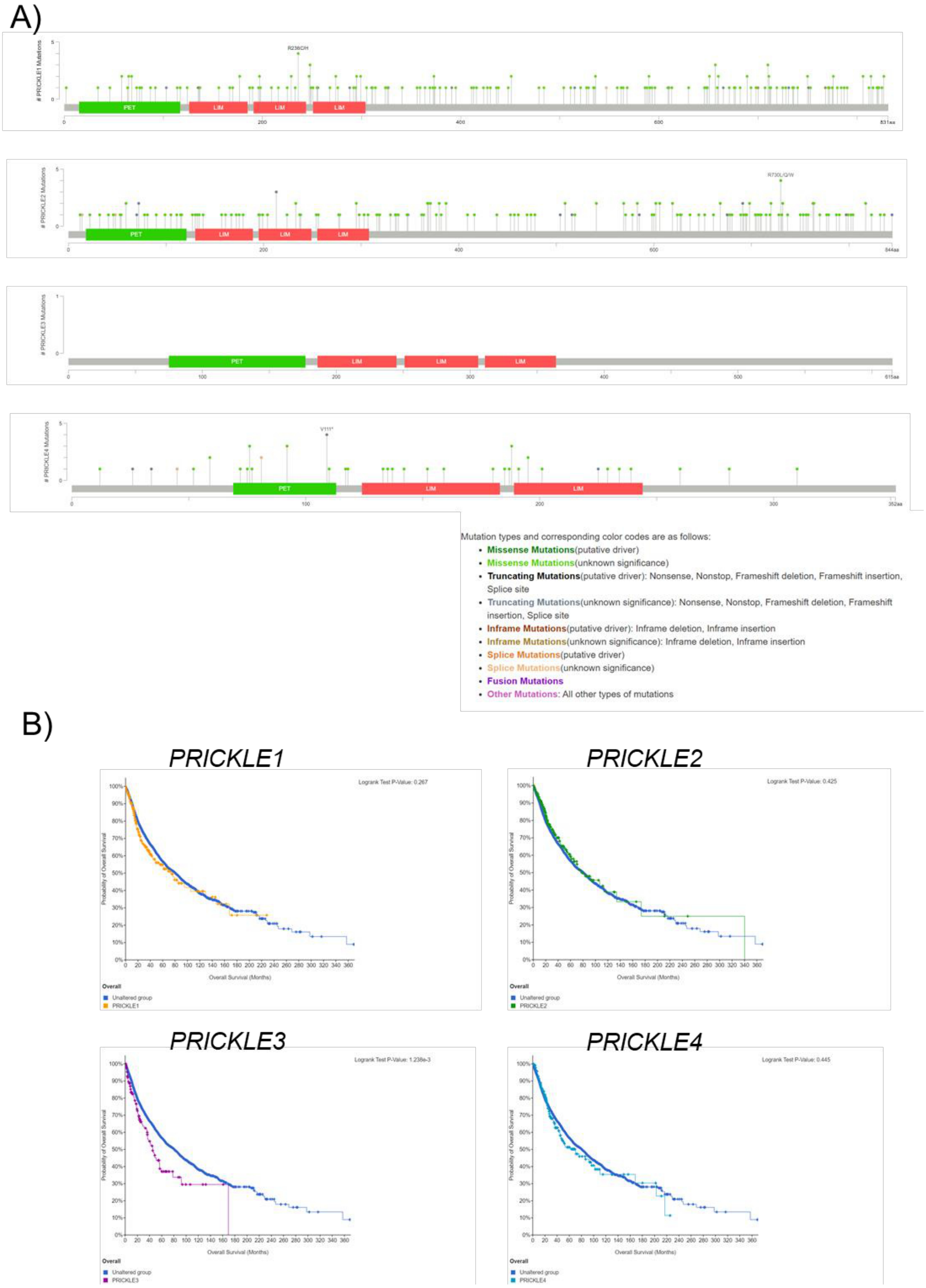

Supplementary Figure 6. **a)** Graphical summary of the nonsynonymous mutations identified in genes encoding PRICKLE isoforms in the TCGA dataset. Legend is shown below the image. **b)** Overall survival of cancer patients with or without germline or somatic mutations in *PRICKLE1-4* genes. Logrank test p-values are present in the graphs.

Supplementary Table 1. The list of TGCA abbreviations

ACC	Adrenocortical carcinoma	LUAD	Lung adenocarcinoma
BLCA	Bladder Urothelial Carcinoma	LUSC	Lung squamous cell carcinoma
BRCA	Breast invasive carcinoma	MESO	Mesothelioma
CESC	Cervical squamous cell carcinoma and endocervical adenocarcinoma	OV	Ovarian serous cystadenocarcinoma
		PAAD	Pancreatic adenocarcinoma
CHOL	Cholangio carcinoma	PCPG	Pheochromocytoma and Paraganglioma
COAD	Colon adenocarcinoma	PRAD	Prostate adenocarcinoma
DLBC	Lymphoid Neoplasm Diffuse Large B-cell Lymphoma	READ	Rectum adenocarcinoma
		SARC	Sarcoma
ESCA	Esophageal carcinoma	SKCM	Skin Cutaneous Melanoma
GBM	Glioblastoma multiforme	STAD	Stomach adenocarcinoma
HNSC	Head and Neck squamous cell carcinoma	TGCT	Testicular Germ Cell Tumors
KICH	Kidney Chromophobe	THCA	Thyroid carcinoma
KIRC	Kidney renal clear cell carcinoma	THYM	Thymoma
KIRP	Kidney renal papillary cell carcinoma	UCEC	Uterine Corpus Endometrial Carcinoma
LAML	Acute Myeloid Leukemia	UCS	Uterine Carcinosarcoma
LGG	Brain Lower Grade Glioma	UVM	Uveal Melanoma
LIHC	Liver hepatocellular carcinoma		
